# Molecular Taxonomy and Diversification of Atlantic Skates (Chondrichthyes, Rajiformes): Adding More Pieces to the Puzzle of Their Evolutionary History

**DOI:** 10.3390/life11070596

**Published:** 2021-06-22

**Authors:** Valentina Crobe, Alice Ferrari, Robert Hanner, Robin W. Leslie, Dirk Steinke, Fausto Tinti, Alessia Cariani

**Affiliations:** 1 Department of Biological, Geological and Environmental Sciences, University of Bologna, 240126 Bologna, Italy; alice.ferrari6@unibo.it (A.F.); alessia.cariani@unibo.it (A.C.); 2 Department of Integrative Biology, University of Guelph, Guelph, ON N1G 2W1, Canada; rhanner@uoguelph.ca; 3 Department of Agriculture, Forestry and Fisheries (DAFF), Branch Fisheries Management, Cape Town 8018, South Africa; roblesliesa@hotmail.com; 4 Department of Ichthyology and Fisheries Science (DIFS), Rhodes University, Grahamstown 6139, South Africa; 5 Department of Integrative Biology, Centre for Biodiversity Genomics, University of Guelph, Guelph, ON N1G 2W1, Canada; dsteinke@uoguelph.ca

**Keywords:** DNA barcoding, phylogenetic inference, skates, Atlantic Ocean, COI, NADH2

## Abstract

Conservation and long-term management plans of marine species need to be based upon the universally recognized key-feature of species identity. This important assignment is particularly challenging in skates (Rajiformes) in which the phenotypic similarity between some taxa and the individual variability in others, hampers accurate species identification. Here, 432 individual skate samples collected from four major ocean areas of the Atlantic were barcoded and taxonomically analysed. A BOLD project ELASMO ATL was implemented with the aim of establishing a new fully available and well curated barcode library containing both biological and molecular information. The evolutionary histories of the 38 skate taxa were estimated with two concatenated mitochondrial markers (COI and NADH2) through Maximum Likelihood and Bayesian inference. New evolutionary lineages within the genus *Raja* were discovered off Angola, where paleogeographic history coupled with oceanographic discontinuities could have contributed to the establishment of isolated refugia, playing a fundamental role among skates’ speciation events. These data successfully resolved many taxonomic ambiguities, identified cryptic diversity within valid species and demonstrated a highly cohesive monophyletic clustering among the order, laying the background for further inference of evolutionary patterns suitable for addressing management and conservation issues.

## 1. Introduction

The world’s fish species are facing a dilemma: on one hand they harbour immense biological richness and ecological function, on the other hand they represent an enormous socio-economic resource. As a consequence of the latter a number of anthropogenic impacts, such as increasingly efficient catching methods, globalization of trade, increasing consumer demand, pollution and habitat loss, are causing alarming declines in both abundance and distribution of many taxa [1,2,3]. Accurate inventories of fish diversity and more reliable species identification at all life-history stages can be addressed through integrated taxonomic approaches and would aid in improved monitoring and help to mitigate the effects of human impacts. However, accurate assessment of species diversity still represents a major challenge for systematic ichthyology [4]. For several taxa, the variability of diagnostic characters among developmental stages is a major impediment to the proper assessment, conservation, and management of global fish biodiversity [5,6,7].

Among elasmobranchs, skates (Order Rajiformes) are the most diverse, with many species exhibiting strongly restricted ranges, leading to a high degree of regional endemism [8,9]. Despite the high number of skate species, their morphology remains highly conserved [10]. In common with other elasmobranchs, they share life-history traits such as a low reproductive potential and a limited capacity for population increase (i.e., slow growth rate, late maturity, low fecundity rate, long gestation periods, and long lifespan [11,12,13]). These reproductive K-strategies make them extremely vulnerable to anthropogenic stressors (e.g., high rate of bycatch) and to over-exploitation [14,15,16,17]. The similar body morphology of most skate species—which is even more evident at early life-history stages [11]—contributes to taxonomic uncertainties and leads to misidentifications in the field that confuse zoogeographic records and negatively impact species assessments. In addition, it allows for mislabelling of seafood products and systematic market substitution [4]. Improvement of identification skills and competency in skate taxonomy is strongly related to the resolution of systematics within Rajiformes [18].

Molecular methods can help to gain more detailed insight into the evolutionary history of chondrichthyans [19] and the prevalence of cryptic species [20,21,22,23], thereby unravelling significant aspects of species diversity in this class of vertebrates. For instance, DNA-based studies on species misidentification and cryptic speciation in skates have become exceedingly common [24,25,26,27]. Genetic analyses of Eastern Atlantic and Mediterranean species of the genus *Raja* helped to clarify relationships among closely related taxa, e.g., the reproductive isolation between *Raja clavata* and *R. straeleni* [28], the taxonomic uncertainties between *R. clavata* and *R. maderensis* [29], the cryptic species complex of *R. miraletus* [30] and the relationship between the Mediterranean endemic skate *R. polystigma* and its sibling species *R. montagui* [31]. Moreover, multiple mitochondrial DNA (mtDNA) markers have been widely used to assess elasmobranch species diversity and phylogeography [32,33] and to estimate levels of divergence and phylogenetic relationships among taxa [34,35,36]. DNA barcoding using the mitochondrial cytochrome oxidase subunit I (COI) fragment [37] has been proven to be a reliable tool for species identification in elasmobranchs [38,39,40,41] and appears to be particularly suited to strengthening morphological taxonomy [42,43,44,45]. For skates, DNA barcodes were used to build species inventories for the North Pacific Ocean [46], the West Atlantic [47,48], the North-East Atlantic [49,50] and the Mediterranean Sea [41].

This study further characterises rajiform species diversity in several areas of the Atlantic Ocean and provides a comprehensive reference library of COI barcodes for the skate species. Moreover, it aims to infer the evolutionary relationships and divergence times of species in relation to historical events in the Atlantic Ocean.

## 2. Materials and Methods

### 2.1. Sampling

**Figure 1:**
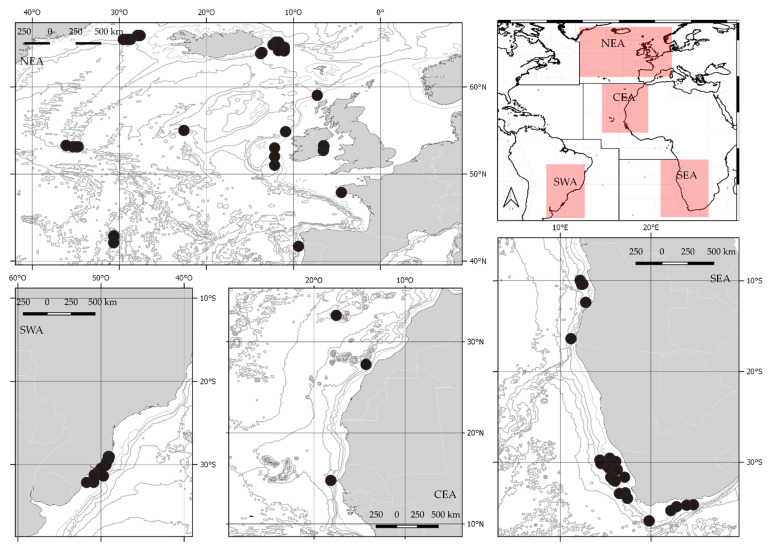
Map of the skate sampling locations in four of the major areas of the Atlantic Ocean: North East Atlantic (NEA), Central East Atlantic (CEA), South East Atlantic (SEA) and South West Atlantic (SWA).

Tissue samples of 462 individual skates were collected during several scientific trawl surveys in the Atlantic Ocean conducted between 2002 and 2011. They cover four major ocean areas: North East Atlantic (NEA), Central East Atlantic (CEA), South East Atlantic (SEA) and South West Atlantic (SWA) (Figure 1). In detail, NEA specimens were collected during different scientific surveys of numerous research projects: BIOICE in 2002–2003 (Iceland) [51]; Bright Sparks in 2007 (Western English Channel) [52]; MAR-ECO during summer 2004 (Mid-Atlantic Ridge) [53]. Additional skate specimens were obtained from landings or fish markets, i.e., the fish markets of Braga (Portugal) and of Concarneau (France). CEA and SEA specimens were caught by three research surveys conducted with the research vessel (R/V) *Dr. Fridtjof Nansen* in Angola in 2006 [54] and R/V *Africana* in South Africa in 2006 and 2011 [55]. SWA specimens were obtained during trawl-fishing cruises in 2006–2007 and deposited in the Museu Oceanográfico do Vale do Itajaí (MOVI).

Skate specimens were identified to species onboard or in the laboratory, using morphological taxonomic characters provided in available identification keys and published guidelines on skate species’ morphology [56,57], resulting in a total of 35 nominal species. Skeletal muscle tissue samples were collected from the body ventral surface and stored in 96% ethanol at −20 °C until molecular analyses.

According to available information on skate distribution [8,58,59] and including recent redescription and resurrection of some taxa [60,61], the four major ocean areas included in this study are home to 92 skate species belonging to 17 genera and three families (Table S1). The IUCN Red List criteria categorized them as Critically Endangered (four), Endangered (eight), Vulnerable (eight), Near Threatened (11), Least Concern (43) and Data Deficient (14). Four species are presently not assessed (Not Evaluated; Table S1).

The ELASMO-ATL project (Project Code: ELATL) was created on the Barcode of Life Data System (BOLD, http://www.boldsystems.org, accessed on 21 March 2021; [62]), and hosts all required fields related to the specimens (identifiers, taxonomy, specimen details, collection data) and, when available, individual dorsal and/or ventral images.

### 2.2. Laboratory Analysis

Total genomic DNA (henceforth gDNA) was extracted from about 20 mg of each sample with the Invisorb^®^ Spin Tissue Mini Kit (Stratec^®^ Molecular, Berlin, Germany) according to the manufacturer’s protocol. A fragment of the mitochondrial cytochrome oxidase subunit I (COI) gene was PCR amplified. One specimen of each taxon identified by COI barcoding was also PCR amplified at the mitochondrial nicotinamide dehydrogenase subunit 2 (NADH2) gene. All PCR reactions were performed in 25 μL of total volume containing 0.625 U of Recombinant Taq DNA polymerase (5 U/µL) PCR kit (Invitrogen, (ThermoFisher Scientific Inc., Monza, Italy), 1× of PCR Buffer, 3 mM of MgCl_2_ and 0.2 mM of dNTP mix. The following cycle conditions were used: an initial DNA denaturation at 95 °C for 2 min followed by 35 cycles at 94 °C for 30 s, 52–55 °C for 30 s, and 72 °C for 1 min, followed by a final extension of 7 min at 72 °C. Primer sequence, final concentration, annealing temperature and literature reference are listed in Table S2. PCR products were enzymatically purified using the ExoSAP-IT™ Express PCR Product Cleanup Reagent (ThermoFisher Scientific Inc., Monza, Italy) and sequencing was performed by a commercial sequence service provider (Macrogen Europe, Amsterdam, the Netherlands). Additional 19 COI sequences of the genus *Dipturus* were retrieved by Carugati et al. in press [63] and were included in the final dataset.

### 2.3. Data Analysis

Trace files were manually checked and edited with the software MEGA X v10.0.1 [64] and sequences were aligned using the ClustalW algorithm [65] implemented in MEGA. The coded amino acidic sequence was assessed to exclude the presence of stop codons and sequencing errors [66]. To rule out any error due to mishandling of samples on board or during the laboratory activities, all sequences were first compared against published sequences via the BOLD Identification Engine and GenBank [67] BLAST (http://blast.ncbi.nlm.nih.gov/Blast.cgi, accessed on 20 November 2020). All COI trace-files and sequence data were uploaded to the ELASMO-ATL project on BOLD.

A Neighbor-Joining (NJ) tree [68] was generated with MEGA using a p-distance metric with pairwise deletion [69]. Bootstrap analysis (BS) with 1,000 replicates [70] was performed to assess statistical support to nodes of the NJ tree topologies. The homologous sequence of *Squalus acanthias* (GenBank code: NC_002012) was used as an outgroup.

Three different algorithms were applied to identify Molecular Operational Taxonomic Units (MOTUs; [71]). The first algorithm applied was the ‘Refined Single Linkage’ (RESL) implemented in BOLD and used to generate Barcode Index Numbers (BINs; [62]). Each BIN was classified as “concordant” or “discordant” with the ‘BIN Discordance Report’ tool implemented on BOLD, depending on whether it comprised sequences attributed to the same nominal species or to different species, respectively. The second method applied was the Bayesian implementation of the Poisson tree processes (bPTP), which is an updated version of the PTP [72], conducted on the web server (available at http://species.h-its.org/ptp/, accessed on 7 May 2021); the parameters for the run were 100,000 MCMC generations, a thinning interval of 100% and 10% of burn-in. The third method applied was the ‘Automatic Barcode Gap Discovery’ (ABGD; [73]) computed on the online web application (http://wwwabi.snv.jussieu.fr/public/abgd/abgdweb.html, accessed on 27 March 2021), using default values.

The maximum intraspecific genetic distance and the mean distance to the Nearest Neighbor (NN) were computed using the ‘Barcoding Gap Analysis’ tool on BOLD [60]. The occurrence of a “barcode gap” [69] was inferred by plotting the maximum intraspecific distance against the mean distance to the NN for each species.

Phylogenetic analyses were performed using single-specimen COI and NADH2 sequences. The homologous sequences of *Squalus acanthias* (NC_002012) were used as outgroups. For a preliminary analysis of the homogeneity of both COI and NADH2 sequence datasets two different approaches were used. The occurrence of alignment ambiguities among taxa was assessed with AliGROOVE v1.06 [74]. Then, the saturation curve of transition and transversion substitutions along with taxa divergence and Xia’s test of substitution saturation [75,76] were carried out in DAMBE v6 [77]. The two fragments were then concatenated using the package SeqinR [78] in R v4.0.4 [79].

The best partitioning of the resulting dataset was inspected using PartitionFinder v2 [80] as implemented in the online CIPRES Science Gateway v3.3 to statistically select the best-fit models of nucleotide substitution through the Bayesian Information Criterion (BIC). The resulting HKY+I+G4 and TRN+I+G4 models for COI and NADH2 datasets respectively, were used to perform the phylogenetic analysis through Maximum-likelihood (ML) and Bayesian Inference (BI) analyses. The ML analysis was conducted through the online implementation of RaxML [81] provided by CIPRES with a bootstrapping (BS) procedure of 1000 replicates. The BI analysis was computed with the software MrBayes v3.2.7 [82] specifying the dataset partitioning and with a MCMC analysis conducted for two runs in parallel with random starting trees, for 500,000 generations and sampled every 5000. Chain was estimated by stable split-standard deviations between the two runs and stable sampled log likelihood values. The posterior probability (prob) of each node was evaluated as a measure of statistical support. All the phylogenetic trees were visualized and edited in FigTree v1.4.4 [83].

## 3. Results

### 3.1. Specimen Identification and Species Delimitation Analyses

A total of 432 skate specimens were successfully barcoded, with a sequence length ranging from 600–674 bp. Runs through the BOLD Identification Engine and BLAST revealed identification errors that occurred either due to mishandling of samples or represented misidentifications: 36 specimens (Table S3) did not match their morphological species identifications. Most of these specimens (N = 29) exhibited 99–100% of COI sequence similarity with matching records. Therefore, they were morphologically reassessed when suitable pictures were available and renamed accordingly, strongly supporting the efficacy of Barcode ID as taxonomic marker in this group. In particular, 10 cases of misidentification occurred between superficially similar species of the same genus (i.e., *Atlantoraja*, *Psammobatis* and *Rajella*); five involved juvenile individuals (e.g., juveniles of *Raja straeleni* misidentified as *Rajella* species); three cases concerning individuals of different genera were probably due to mislabelling of samples in the field as morphological assessment from images confirmed molecular identification. Unfortunately, neither images nor voucher specimens are available for 11 specimens and morphological re-evaluation was not possible. Identification of these 11 specimens was reassigned following the Barcode ID. The remaining seven COI sequences, belonging to *Raja* individuals collected off Angola, showed lower similarity values to *Raja herwigi* (98.77%) and *R. clavata* (98.44%) (Table S3). The weak similarity did not allow a robust and definitive molecular species identification of these seven individuals, which were temporarily assigned to *Raja sp*. and were considered as a new COI lineage of the complex *R. clavata*/*R. maderensis*/*R. straeleni*.

**Figure 2:**
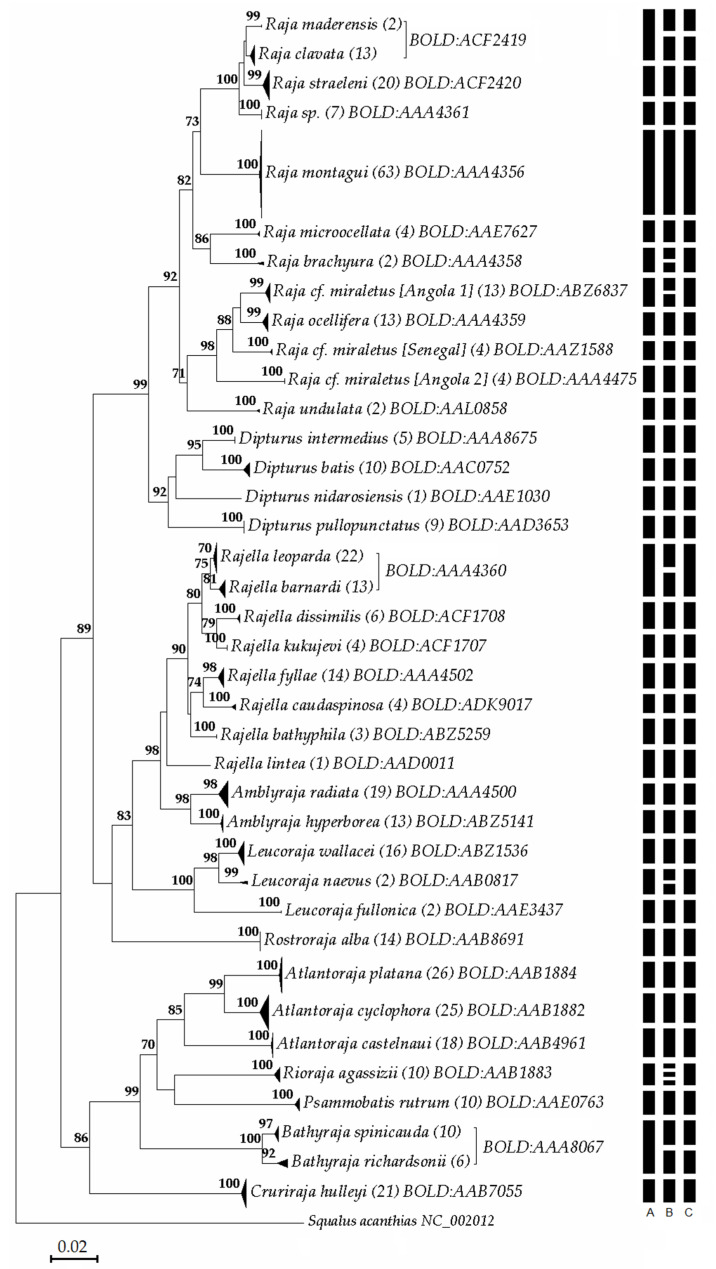
Neighbor-Joining tree based on COI genetic p-distances of Atlantic skate taxa. Numbers near nodes indicate bootstrap values (≥ 70%). Numbers in parentheses indicate the number of individuals analysed for each clade. A distance scale bar is given. The results of species delimitation analyses using RESL (A), bPTP (B) and ABGD (C) algorithms respectively, are shown as vertical bars on the right.

The NJ tree (Figure 2) shows that most of the species-specific clusters obtained are supported by BS values >70%, except for *Raja clavata*, with a BS of 69%. The *R. miraletus* species complex split into four fully supported geographical sub-clusters: one formed by samples collected from Senegal, two from the Angola and one from South Africa (corresponding to the resurrected *R. ocellifera*). 

The three MOTU delimitation approaches used yielded slightly different results (Figure 2). The RESL algorithm identified 35 BINs (Table S4; Figure 2A). The BIN discordance analysis categorized three BINs as discordant with a taxonomic rank of conflict between congeneric species of Bathyraja, *Raja* and *Rajella* (Table S4): the BIN BOLD:AAA8067 included 10 *Bathyraja spinicauda* and six *B. richardsoni*, the BIN BOLD:ACF2419 included 14 individuals of *Raja clavata* and two of *Raja maderensis* and the BIN BOLD:AAA4360 included 13 sequences of *Rajella barnardi* and 22 of *Rajella leoparda.*

The remaining 32 BINs were concordant since they included only specimens of the same species. Five of the BINs were classified as “Unique BINs” because they included records belonging to species barcoded ex novo: two of them were associated to newly barcoded species (*Psammobatis rutrum* and *Rajella caudaspinosa*), whereas the other two unique BINs were associated with *Raja miraletus*. Lastly, BIN BOLD:AAA4361 was associated with the seven specimens from Angola herein assigned to *Raja sp*. bPTP identified 43 MOTUs, three of these were attributed to the same taxon *Rioraja agassizii*, two to *Leucoraja naevus*, two to *Raja brachyura* and two to *Raja* cf. *miraletus* [Angola 1]; all the others corresponded to unique nominal species (Figure 2B). Finally, the ABGD method identified 37 MOTUs corresponding to unique nominal species except for *Rajella leoparda* and *Rajella barnardi* (Figure 2C).

The maximum p-distance within taxa was 0.61 on average (range = 0–1.20, Table S4) with the highest value occurring in *Amblyraja radiata*. The lowest genetic distances to the NN were attributed to *Raja leoparda/Raja* *barnardi* (0.85%) followed by *Raja clavata*/*Raja* *maderensis* (0.92%). In BOLD, species pairs with <2% of genetic distances are marked as potential problematic taxa and this also occurred for the pairs *Raja* *clavata* and *Raja* *straeleni* (1.20%), *Rajella dissimilis* and *Rajella kukujevi* (1.20%), *Bathyraja richardsoni* and *B*. *spinicauda* (1.55%), *Raja sp*. and *Raja* *clavata* (1.72%), *Rajella caudaspinosa* and *Rajella fyllae* (1.89%). The maximum intraspecific p-distance was always lower than the distance to the NN as showed in Figure S1.

Overall, the final assessment of 432 sequences showed a total of 38 MOTUs and 35 nominal species. According to IUCN categories, our data included two Critically Endangered species, five Endangered, five Vulnerable, five Near Threatened, 16 Least Concern, one Data Deficient and one Not Evaluated species (Table S4).

### 3.2. Phylogenetic Inference and Divergence Time Estimates

Due to the homogeneity of both the COI and NADH2 datasets and the unsaturated numbers of transition and transversions (Figures S2 and S3; Table S5), the development of a concatenated sequence dataset of the two mitochondrial markers was considered justified, yielding a sequence dataset, with a total length of 1277 bp (NADH2: 677 bp and COI: 600 bp) was build.

Phylogenies inferred from ML and BI were partially consistent and highly supported by bootstrap values and posterior probabilities, respectively (Figure S4). Three major monophyletic lineages corresponding to the three skate families Rajidae, Gurgesiellidae and Arhynchobatidae were obtained. Rajidae was placed as sister taxon to the other two families and Gurgesiellidae (represented by a single species *Cruriraja hulleyi*) was the sister taxon of Arhynchobatidae. Within Rajidae, three major subclades can be distinguished corresponding to the tribes (i) Amblyrajini, comprising the taxa *Rajella*, *Amblyraja* and *Leucoraja*; (ii) Rajini, with *Raja* and *Dipturus*, and (iii) Rostrorajini, represented by a single species *Rostroraja alba*. Within Arhynchobatidae, three subclades/tribes can be identified: (iv) Arhynchobatini represented by *Psammobatis rutrum,* (v) Bathyrajini represented by *Bathyraja* species and (vi) Riorajini with *Atlantoraja* and *Rioraja*. All congeneric species clustered monophyletically in both ML and BI topologies.

## 4. Discussion

### 4.1. ELASMO-ATL: A Reference COI Library for Atlantic Skates

The ELASMO-ATL project confirmed the usefulness and effectiveness of DNA barcoding for the taxonomy and identification of Atlantic skates. The effectiveness of DNA barcoding was demonstrated by cohesive monophyletic clustering, the high rate of concordant BINs/MOTUs and by the presence of a “barcode gap” between intraspecific and interspecific variability for all taxa analysed. Nevertheless, success in species identification using COI barcoding may be influenced by geographical scale: higher levels of intraspecific distance and lower distance to Nearest Neighbor are expected with increasing geographical sampling range [84].

The integration of ELASMO-ATL results with those of the other barcoding initiatives carried out in the North East Atlantic [49,50,85,86,87] raises the total count to 28 North Atlantic barcoded skates, now including the two species *Bathyraja richardsoni* and *Rajella bathyphila*, previously barcoded only in the North West Atlantic [47] (see Table S1). The only two species without a barcode in this area, *Malacoraja kreffti* and *M. spinacidermis*, have been barcoded from the West Atlantic [43]. ELASMO-ATL expands the molecular taxonomic coverage of the South East Atlantic, now comprising 16 of the 22 species reported from this large area. This includes *ex novo* barcodes for *Rajella caudaspinosa*. Four species with available COI sequences, *Cruriraja hulleyi, Raja ocellifera, Rajella barnardi* and *Rajella leoparda,* were not reported in the literature before, thus ELASMO-ATL serves as reference for barcode data of these species. Among the species not targeted by ELASMO-ATL, only *Bathyraja smithii* and *Dipturus springeri* have already been barcoded in this area [88]. In the South West Atlantic, five out of 39 known species were recorded, with specimens from a single Brazilian campaign in 2007. One of these species, *Psammobatis rutrum*, was barcoded ex novo in the present study. Most of the South West Atlantic skates were barcoded by prior initiatives in Argentinian and Antarctic waters [38,48,89,90]. Due to the limited sampling of the Central-Western African coasts, it is not possible to assess within-species genetic variation along the West African coast. The lack of samples from several geographic areas, such as the northwestern Atlantic Ocean, and the uneven sampling effort across the considered four major ocean areas (which resulted in different coverage both on the geographical and bathymetric range) is an inherent flaw and limit of our study. This limit is a common issue of research studies on Elasmobranch taxa which are not targets of commercial fishing or dedicated scientific surveys. Integration and coordination of research efforts under a shared framework such as BOLD represents an opportunity to overcome these gaps. Pronounced stasis of gross body morphology and species-specific characters exhibited prevalently in adults [23] caused relevant constraints on morphological taxonomy of elasmobranchs. Most of the skates collected incidentally during international trawl surveys, especially surveys aiming at estimating recruitment of commercially important fish species, are juveniles or sub-adults [91]. Current field guides and taxonomic keys often rely on characters visible only in adults and can be ineffective in the identification of younger life stages. This can compromise the collection of robust scientific data on species-specific distribution and fishing mortality. Although DNA barcodes can help to identify and distinguish species, they can’t resolve all the issues surrounding species delineation. Thus, they should be used in combination with other molecular markers [92] and other traits such as ecology and morphology [18]. The field-based species misidentifications detected in the present study were most likely linked to various factors such as: close morphological similarity (e.g., species in the genus *Rajella*-*R. barnardi*, *R. dissimilis*, *R. leoparda*; [59]); relying on a single identification character (e.g., thick spiny tail shared by *Cruriraja hulleyi* and *Rajella caudaspinosa* and spotted disc shared by *Rajella leoparda* and juvenile *C. hulleyi*). Finally, misidentification of species that are morphologically clearly distinguishable (e.g., *L. wallacei* and *Raja straeleni*) may be the result of mishandling or mislabelling of tissue samples.

Although the molecular species delimitation analysis clearly supported different valid species, most of the pairwise estimates of genetic divergence between species (especially within *Rajella*) yielded values <2%. Even if species pairs with genetic distances below this threshold are highlighted as potential problematic taxa in BOLD, Hebert et al. [37] suggested that the best threshold should be estimated according to the target taxon. In fact, it has been demonstrated that nucleotide substitution rate in cartilaginous fishes is slower than in other vertebrate taxa [93,94,95]. Therefore, we suggest that this empirical cut-off may not be effective for elasmobranchs in general and for skates in particular. A fixed gap value would imply a non-evolving feature of the species, which is highly unlikely [96,97,98], therefore the distance to the Nearest Neighbor species could be used as a standard measure to maintain conservative estimates. All of the MOTU delimitation approaches (except bPTP analysis) failed to differentiate between the two most common deep–sea skate species found off the west coast of southern Africa [55], *R. barnardi* and *R. leoparda*. In contrast, the maximum value of intraspecific p-distance was lower than the distance to the Nearest Neighbor highlighting the presence of a “barcode gap”. These sister taxa are characterized by a sympatric distribution and a very recent common ancestor. The lack of divergence at the mtDNA loci between putative species may be the result of a paucity of polymorphisms. With very recent separation there may have been insufficient time (number of generations) for reciprocal monophyly to have developed. This could be the case of the recently diverged “*Raja clavata*” clade of sibling taxa, *R. straeleni*, *R. clavata* and *R. maderensis* that is taxonomically resolved only by the ABGD method. Previous studies have reported difficulties in separating sibling species of skates: Serra-Pereira et al. [49] failed to determine whether *Raja clavata* and *R. maderensis* really constitute distinct species when using COI sequences; subsequently Ball et al. [29] studied the same two species and failed to support the genetic distinctiveness using a combined analysis of both the Control Region and COI. Coulson et al. [47] had difficulties to separate *Amblyraja jenseni* and *A. badia* from *A. hyperborea* and subsequently they were considered possibly conspecific by Weigmann [8]. *Raja montagui* could not be genetically differentiated from the Mediterranean *R. polystigma* [31] due to the occurrence of natural hybridization between the two species in sympatric areas. Finally, the COI analysis conducted by Spies et al. [46] in the Bering Sea, could not differentiate between *Bathyraja* *matsubarai* and *B. maculata.* The inability to discriminate between *Rajella barnardi* and *R. leoparda* reported here may be resolved by usingsequence markers that evolve more rapidly (e.g., mitochondrial Control Region or nuclear intronic sequences). All aforementioned data representing cases where barcoding failed to demonstrate differentiation between morphologically recognized species, highlights the limits of using a single marker gene as molecular tool [69], but on the other hand they indicated that in skates the recent steps of the evolutionary animation leading to speciation could be characterized by low levels of molecular and morphological divergence.

### 4.2. Paleogeographic History and Oceanographic Discontinuities has been Driven Diversification of Atlantic Skates

Most phylogenetic relationships among skate taxa were strongly supported by both ML and BI analyses and are concordant with those obtained by previous studies based on mtDNA [10,34,36], combined mtDNA and nuclear DNA markers [28,35], and mitogenomes [99,100]. Phylogenetic trees clustered skate taxa into seven main lineages corresponding to the tribes Rajini (e.g., *Raja* and *Dipturus*), Amblyrajini (e.g., *Amblyraja*, *Leucoraja* and *Rajella*), Rostrorajini (e.g., *Rostroraja*), Crurirajini (e.g., *Cruriraja*), Arhynchobatini (e.g., *Psammobatis*), Bathyrajini (e.g., *Bathyraja*) and Riorajini (e.g., *Rioraja* and *Atlantoraja*). This topology of skate evolution broadly corresponds to that obtained from the fundamental cladistic analysis of internal and external morphological characters by McEachran and Dunn [101]. A relevant uncertainty is the phylogenetic position of the tribe Rostrorajini. Previous morphological observations placed *Rostroraja alba* within the Rajini [9,102] while other phylogenies inserted the species into the Amblyrajini [29,34,36,103]. Our phylogeny is consistent with the phylogeny presented by Chiquillo et al. [104], who elevated the Rostrorajini to the tribe level and placed it as a sister group of the other two tribes within the Family Rajiidae. Moreover, the morphologically-based reallocation of *Dipturus linteus* within the genus *Rajella* [104] was supported by both ML and BI phylogenies, although the genus *Rajella* remains poorly supported in the ML tree (BS < 0.50; Figure S4), but highly supported in the BI reconstruction (prob = 0.99; Figure S4). The phylogenetic position of *Raja undulata* as the basal taxon of *Raja* in the ML tree was consistent with the recent molecular phylogeny inferred by Kousteni et al. [100].

Meanwhile the increasing application of molecular techniques and the finding of divergent mtDNA lineages within currently valid skate species are leading to the discovery of a huge amount of cryptic diversity in this marine group exhibiting non-visual mate recognition system (see Bickford et al. [105]). The widely distributed *Raja miraletus* complex may provide an exciting new case: five deeply distinct mtDNA lineages were identified in this apparently morphologically-frozen taxon that has been evolving since the Middle Miocene in the Atlantic and Mediterranean. The southernmost lineage occurs off South Africa and corresponds to the recently resurrected *R. ocellifera* [30], previously reported as ‘*Raja* cf. *miraletus 1*’ using the NADH2 gene by Naylor et al. [34]. The Senegalese lineage, here reported as ‘*Raja* cf. *miraletus* [Senegal]’, corresponds to an as yet undescribed taxon [30] that was previously identified as *Raja miraletus* by Naylor et al. [34] and co-occurs in Senegal and Mauritania with a species recently described by Last and Séret [30] (*R. parva*, not included in the present study). Two new lineages were identified from the Angolan coast: ‘*Raja* cf. *miraletus* [Angola 1]’, and ‘*Raja* cf. *miraletus* [Angola 2]’. This complicated Atlantic evolutionary scenario is further enriched by the northernmost lineage *R. miraletus*, occurring in the Mediterranean Sea and adjacent North Eastern Atlantic waters [33]. It was, however, not included in this study. Further genetic analyses that overcome the limitations of mtDNA in assessing reproductively isolated units instead of evolutionary lineages are needed to solve this intricate evolutionary history. Similarly, a new ancient Angolan lineage was identified within the clade formed by *Raja straeleni/R. clavata/R. maderensis*. *R. clavata* has a prevalent North-Eastern Atlantic and Mediterranean range [58]. In contrast to the congeneric *R. miraletus* group, which shows high levels of morphological stasis, the general morphology of *R. clavata* is noticeably variable and considered polytypic [106], leading Ball et al. [29] to consider *R. maderensis* as a morphological variant of the thornback ray. The southernmost part of the clade’s distribution is the biscuit skate *R. straeleni*, endemic to the South-Eastern Atlantic and Western Indian Ocean. *Raja straeleni* appears to be closely related to *R. clavata*, from which it diverged in the late Pliocene/early Pleistocene [28]. A lack of specimens representing *Raja clavata*-like species such as *R. herwigi* from the Central-Eastern Atlantic prevents a comprehensive assessment of the taxonomic status of the ancient Angolan lineage of this group, reported here and referred as *Raja sp.*, and the level at which diversification of lineages occurred.

All these sister taxa can be considered the result of the synergistic influence of current systems and upwelling zones of the central western African waters, which may have driven multiple speciation processes and led to relevant richness of marine species diversity. (1) The Senegalese-Mauritanian upwelling zone (12° N–20° N) and the Canary current system [107] act as a physical and thermal barrier to southwards dispersal of taxa with cold-temperate affinities. They constitute the northern limit of species with subtropical-tropical distribution [108]. In this region, the specific features of Cape Verde and the Canary Islands linked to their volcanic origin, insularity and oceanographic conditions could have influenced the diversification of the Senegalese skate taxa, following the important change of marine biodiversity in the area [109,110]. (2) The Benguela upwelling system, extending from South Africa to Angola [111], forms a hydro-biological barrier for many species [55]. The Angola-Benguela front, at about 16–18° S, constitutes a thermal barrier between the northern oligotrophic tropical waters and the southern upwelled cold, nutrient-rich waters [112,113]. This oceanographic system would have likely reduced the gene flow between the Angolan and the South African skate taxa, which was confirmed as a driving factor for the evolution of other South-Eastern Atlantic fish [114,115].

## 5. Conclusions

ELASMO-ATL, a well-curated COI barcode library is presented and provided as a referenced catalogue of species of Rajiformes in Atlantic waters, encouraging the collection of natural history data over a broad area and providing specimens and corresponding reference sequences for future studies. DNA barcoding solved a number of cases of misidentification errors within Rajiformes and uncovered new evolutionary lineages. In view of the alarming declines in abundance and distribution of many fish species, and the population collapse observed for many large Chondrichthyan species, correct species identification is essential for conservation and long-term management. Given the overall similarity in body-plan similarity and gross morphology among skate species, detailed taxonomic expertise is required for accurate species identification at sea. More accurate information from scientific surveys and landing data is essential to improve the assessment of spatial and temporal patterns of skate species diversity in the Atlantic Ocean.

The analyses of phylogenetic relationships among Atlantic skates allowed us to hypothesize the plausible natural history of several taxa. We assumed that ancient climate changes and oceanographic discontinuities played a key role in the diversification of recent *Raja* lineages of the Central and South East Atlantic Ocean. Uncertainties remain with respect to the driving forces responsible for the recent divergence of *Rajella barnardi* and *R. leoparda*, two endemic sympatric South Atlantic skates.

## Data Availability

Sampling and biological data, digital images of dorsal/ventral sides (when recorded) as well as COI and NADH2 sequences are available in the “ELASMO-ATL” project (Project Code: ELATL) accessible on the Barcode of Life Data system (BOLD, http://www.barcodinglife.org, accessed on 22 June 2021).

## Supplementary Materials

The following are available online at https://www.mdpi.com/article/10.3390/life11070596/s1, Table S1. Complete list of skate species occurring in the North East (NEA), Central East (CEA), South East (SEA) and South West (SWA) Atlantic Ocean. References related to the bar-code data and the last global assessment by IUCN are included. LC: least concern, NT: near threatened, VU: vulnerable, EN: endangered, CR: critically endangered, NE: not evaluated. Grey highlights species and geographical areas covered by this study. Table S2. Primer sequences in 5′-3′ direction, final concentration (Cf, in mM) and annealing temperature (Ta, in °C) utilised in this study for PCR amplification of the two partial mitochondrial fragments Cytochrome Oxidase subunit I (COI) and Nycotinamide dehydrogenase subunit 2 (NADH2). Grey boxes mark the M13-sequencing primer. Table S3. Cases of specimen misidentification. Percentage similarity (through BOLD Identification Engine) and sampling area are presented; the number of misidentified individuals in relation to the total number assessed is provided in parenthesis in the Morphological ID and Barcode ID column. Table S4. Sequence diversity for the Cytochrome Oxidase subunit 1 of Atlantic skates included in the present study. For each taxon, the following fields are presented: number of individuals (N), maximum intraspecific genetic distance (Max Intra-Sp), distance to Nearest Neighbor (Distance to NN), number of haplotypes (nH), sampling locations, Barcode Index Number (BIN) obtained from BOLD (blue text refers to Unique BIN, red text refers to Discordant BIN, black text refers to Concordant BIN), and abbreviation ID used in phylogenetic analyses. IUCN Categories are also indicated: least concern (LC), near threatened (NT), vulnerable (VU), endangered (EN), critically endangered (CR), not evaluated (NE). Table S5. Substitution saturation test measuring whether the observed index of substitution saturation (Iss) is significantly lower than critical Iss value (Iss.c), with different numbers of operational taxonomic units (Num.OTU) for COI and NADH2. Figure S1. Maximum intraspecific distance plotted against the minimum inter-specific (Nearest Neighbor) distances (p-distance values) for the COI barcode sequences of the 38 MOTUs. The 1:1 equivalence (straight line) is indicated. Figure S2. AliGROOVE analysis based on 38 mitochondrial sequences of Atlantic skates. (a) NADH2; (b) COI. The observed mean similarity score between sequences is represented by the colour of the square. Taxon names are consistent with the column “ID” in Table S4. Figure S3. Plot of the number of transitions (s, blue cross) and transversions (v, green triangle) vs. genetic distance. The curves show the trends of variance of transitions and transversions with increasing genetic distance. (a) NADH2; (b) COI. Figure S4. Maximum-likelihood (a) and Bayesian (b) phylogenetic trees based on concatenated NADH2 and COI datasets. Statistical measures of Maximum-likelihood bootstrap (BS) and Bayesian posterior probability (prob) supports are shown at each node. A distance scale bar is given. Names at tips are consistent with the column “ID” in Table S4.
