# Supplementary material for: Molecular Taxonomy and Diversification of Atlantic Skates (Chondrichthyes, Rajiformes): Adding More Pieces to the Puzzle of Their Evolutionary History
Source: Life (Basel). 2021 Jun 22;11(7):596. doi: 10.3390/life11070596 (PMC8303890; doi:10.3390/life11070596)
Supplement: Supplementary file 1 [file life-11-00596-s001.zip › life-1252447-supplementary.pdf]

life

MDPI

Supplementary material of Molecular Taxonomy and Diversification of Atlantic Skates (Chondrichthyes, Rajiformes): Adding more Pieces to the Puzzle of Their Evolutionary History

Table S1. Complete list of skate species occurring in the North East (NEA), Central East (CEA), South East (SEA) and South West (SWA) Atlantic Ocean. References related to the barcode data and the last global assessment by IUCN are included. LC: least concern, NT: near threatened, VU: vulnerable, EN: endangered, CR: critically endangered, NE: not evaluated. Grey highlights species and geographical areas covered by this study.

Scientific Name (Author/s, Year)	Common Name	NEA	CEA	SEA	SWA	Barcoded in the area	Barcoded in other areas	References	Global IUCN Last assessed	
Amblyraja doellojuradoi (Pozzi, 1935)	Southern thorny skate				x	yes	no	[1]	LC	2019
Amblyraja frerichsi [1] (Krefft, 1968)	Thickbody skate				x	no	no		DD	2004
Amblyraja georgiana [1] (Norman, 1938)	Antarctic starry skate				x	yes	no	Unpublished	DD	2019
Amblyraja hyperborea (Collett, 1879)	Arctic skate	x		x		yes	yes	[2,3]	LC	2016
Amblyraja jenseni [1] (Bigelow & Schroeder, 1950)	Shorttail skate	x				yes	no	[2]	LC	2019
Amblyraja radiata (Donovan, 1808)	Starry ray	x				yes	yes	[2–5]	VU	2019
Amblyraja taaf [1] (Meisner 1987)	Whiteleg skate				x	no	no	[8]	DD	2020
Atlantoraja castelnaui (Miranda Ribeiro, 1907)	Spotback skate				x	yes	no	[1,6,7]	CR	2019
Atlantoraja cyclophora (Regan, 1903)	Eyespot skate				x	yes	no	[1,6]	EN	2019
Atlantoraja platana (Günther, 1880)	La Plata skate				x	yes	no	[1,6]	EN	2019
Bathyraja albomaculata (Norman, 1937)	White-dotted skate				x	yes	no	[1]	VU	2019
Bathyraja brachyurops (Fowler, 1910)	Broadnose skate				x	yes	no	[1,4]	NT	2019
Bathyraja cousseauae Díaz de Astarloa & Mabragaña, 2004	Joined-fins skate				x	yes	no	[1]	LC	2019
Bathyraja griseocauda (Norman, 1937)	Graytail skate				x	yes	no	[1]	EN	2019
Bathyraja hesperafricana Stehmann, 1995	West African skate		x			no	no		DD	2004
Bathyraja macloviana (Norman, 1937)	Patagonian skate				x	yes	no	[1]	NT	2019
Bathyraja magellanica (Philippi, 1902)	Magellan skate				x	yes	no	[1]	LC	2019
Bathyraja meridionalis Stehmann, 1987	Dark-belly skate				x	yes	no	[8]	LC	2019
Bathyraja multispinis (Norman, 1937)	Multispine skate				x	yes	no	[1,4]	NT	2019
Bathyraja pallida (Forster, 1967)	Pale ray	x				yes*	no		LC	2019

Bathyraja papilionifera Stehmann, 1985	Butterfly skate				x	yes	no	[1]	LC	2019
Bathyraja richardsoni (Garrick, 1961)	Richardson's ray	**x**				this study	yes	[8,9]	LC	2015
Bathyraja scaphiops (Norman, 1937)	Cuphead skate				x	yes	yes	[1]	LC	2019
Bathyraja schroederi(Krefft, 1968)	Whitemouth skate				x	no	no		LC	2019
Bathyraja smithii (Müller & Henle, 1841)	African softnose skate			x		yes	no	Unpublished	LC	2019
Bathyraja spinicauda (Jensen, 1914)	Spinytail skate	**x**				yes	no	[2,3]	NT	2019
Cruriraja durbanensis (von Bonde & Swart, 1923)	Smoothnose legskate			x		no	no		DD	2019
Cruriraja hulleyi Aschliman, Ebert & Compagno, 2010	Roughnose legskate			**x**		yes	no	Unpublished	LC	2019
Dactylobatus clarkii (Bigelow & Schroeder, 1958)	Hookskate				x	no	no		LC	2019
Dipturus argentinensis Díaz de Astarloa, Mabragaña, Hanner & Figueroa, 2008	Argentine Skate				x	yes	no	[1]	DD	2019
Dipturus batis (Linnaeus, 1758)	Blue skate	**x**				yes	no	[5,10,11]	CR	2006
Dipturus doutrei (Cadenat, 1960)	Violet skate		x	x		no	no		DD	2009
Dipturus intermedius (Parnell, 1837)	Flapper skate	**x**				yes	no	[3]	NE	-
Dipturus lamillai Concha, Caira, Ebert & Pompert, 2019	Warrah Skate				x	no	no		LC	2020
Dipturus leptocaudus (Krefft & Stehmann, 1975)	Thintail skate				x	no	no		VU	2019
Dipturus mennii Gomes & Paragó, 2001	South Brazilian skate				x	no	no		CR	2019
Dipturus nidarosiensis (Storm, 1881)	Norwegian skate	x	**x**	x		yes	yes	[3,4,10,12–15]	NT	2015
Dipturus oxyrinchus (Linnaeus, 1758)	Longnosed skate	x	x			yes	yes	[3,4,10,11,13, 16–19]	NT	2014
Dipturus pullopunctatus (Smith, 1964)	Slime skate			**x**		yes	no	[4,20]	LC	2020
Dipturus springeri (Wallace, 1967)	Roughbelly skate			x		yes	no	[4,20]	LC	2019
Dipturus teevani (Bigelow & Schroeder, 1951)	Prickly brown ray				x	no	no		LC	2019
Dipturus trachydermus (Bigelow & Schroeder, 1950)	Roughskin Skate				x	no	no		EN	2019
Gurgesiella dorsalifera McEachran & Compagno, 1980	Onefin skate				x	no	no		VU	2019
Leucoraja circularis (Couch, 1838)	Sandy ray	x	x			yes	yes	[3,11,13,16,19]	EN	2014
Leucoraja compagnoi (Stehmann, 1995)	Tigertail skate			x		no	no		DD	2019
Leucoraja fullonica (Linnaeus, 1758)	Shagreen ray	**x**				yes	yes	[3]	VU	2014
Leucoraja leucosticta (Stehmann, 1971)	Whitedappled skate		x			no	no		DD	2018
Leucoraja naevus (Müller & Henle, 1841)	Cuckoo ray	**x**				yes	no	[4,5,11,13,16, 19]	LC	2014
Leucoraja wallacei (Hulley, 1970)	Yellowspotted skate			**x**		yes	no	[20]	VU	2020

Malacoraja kreffti (Stehmann, 1978)	Krefft's ray	x				no	yes	Unpublished	LC	2014
Malacoraja obscura Carvalho, Gomes & Gadig, 2005	Brazilian Soft Skate				x	no	no		LC	2019
Malacoraja spinacidermis (Barnard, 1923)	Soft skate	x	x	x		no	yes	[2]	LC	2020
Neoraja africana (Stehmann & Séret, 1983)	West African pygmy skate		x			no	no		DD	2006
Neoraja caerulea (Stehmann, 1976)	Blue ray	x				yes	no	[12]	LC	2014
Neoraja iberica Stehmann, Séret, Costa & Baro, 2008	Iberian pygmy skate	x				yes	no	[12,16]	LC	2014
Neoraja stehmanni (Hulley, 1972)	South African Dwarf Skate			x		no	no		LC	2019
Psammobatis bergi Marini, 1932	Blotched sandskate				x	yes	no	Unpublished	LC	2019
Psammobatis extenta (Garman, 1913)	Zipper sandskate				x	yes	no	Unpublished	LC	2019
Psammobatis lentiginose McEachran, 1983	Freckled sandskate				x	yes	no	[1]	LC	2019
Psammobatis normani McEachran, 1983	Shortfin sandskate				x	yes	no	[1]	LC	2019
Psammobatis parvacauda McEachran, 1983	Smalltail sandskate				x	no	no		DD	2007
Psammobatis rudis Günther, 1870	Smallthorn sandskate				x	yes	no	[1]	LC	2019
Psammobatis rutrum Jordan, 1891	Spade sandskate				**x**	this study	no		LC	2019
Raja asterias Delaroche, 1809	Mediterranean starry ray	x				yes	yes	[13,19]	NT	2014
Raja brachyura Lafont, 1871	Blonde ray	**x**	x			yes	yes	[3,5,11,13,16, 17,19]	NT	2008
Raja clavata Linnaeus, 1758	Thornback ray	**x**	x	x		yes	yes	[3–5,11,13,16–19,21]	NT	2005
Raja herwigi Krefft, 1965	Cape Verde skate		x			yes*	no	Unpublished	DD	2007
Raja maderensis Lowe, 1838	Madeira skate		**x**			yes	no	[16,22]	VU	2014
Raja mauritaniensis [2] Capapé, 1977	African ray		x			no	no		NE	-
Raja microocellata Montagu, 1818	Small-eyed ray	**x**	x			yes	no	[5,16]	NT	2006
Raja miraletus Linnaeus, 1758	Brown ray	x	**x**			yes	yes	[4,11,13,16–19,21]	LC	2019
Raja montagui Fowler, 1910	Spotted ray	**x**	x			yes	yes	[3–5,11,13,16,19]	LC	2007
Raja ocellifera Regan, 1906	Twineye skate			**x**		yes	no	Unpublished	EN	2020
Raja parva Last & Séret, 2016	African brown skate		x	x		yes**	no	[23]	NE	-
Raja straeleni Poll, 1951	Biscuit skate		x	**x**		yes	no	[20]	DD	2009
Raja undulata Lacepède, 1802	Undulate ray	**x**	x			yes	yes	[13,16]	EN	2003

Rajella barnardi (Norman, 1935)	Bigthorn skate		x	**x**		yes	no	Unpublished	LC	2004
Rajella bathyphila (Holt & Byrne, 1908)	Deep-water ray	**x**	x			this study	yes	[2]	LC	2019
Rajella bigelowi (Stehmann, 1978)	Bigelow's ray	x	x			yes	yes	Unpublished	LC	2019
Rajella caudaspinosa (von Bonde & Swart, 1923)	Munchskin skate			**x**		this study	no		LC	2019
Rajella dissimilis (Hulley, 1970)	Ghost skate		x	**x**		yes	no	[4,20]	LC	2004
Rajella fyllae (Lütken, 1887)	Round ray	**x**				yes	yes	[2,3]	LC	2019
Rajella kukujevi (Dolganov, 1985)	Mid-Atlantic skate	**x**				yes	no	[12]	LC	2014
Rajella leoparda (von Bonde & Swart, 1923)	Leopard skate		x	**x**		yes	no	Unpublished	LC	2018
Rajella lintea (Fries, 1838)	Sailray	**x**				yes	yes	[3]	LC	2019
Rajella ravidula (Hulley, 1970)	Smoothback skate		x	x		no	no		LC	2019
Rajella sadowskii (Krefft & Stehmann, 1974)	Brazilian skate				x	no	no		DD	2004
Rioraja agassizii (Müller & Henle, 1841)	Rio skate				**x**	yes	no	[1,7]	VU	2019
Rostroraja alba (Lacepède, 1803)	White skate	x	x	**x**		yes	yes	[13,16,18–20]	EN	2006
Sympterygia acuta Garman, 1877	Bignose fanskate				x	yes	no	[1]	CR	2019
Sympterygia bonapartii Müller & Henle, 1841	Smallnose fanskate				x	yes	no	[1]	NT	2019
Zearaja brevicaudata (Marini, 1933)					x	yes	no	[27]	NE	-

[1] These species were considered possibly conspecific with *Amblyraja hyperborea* by Weighman [28] and treated as junior synonym of *A. hyperborea* by Last et al. [29].

[2] *Raja mauritaniensis* was proposed as a replacement name for the junior homonym *Raja Africana* by White & Fricke [30]. The species had previously been treated as a questionably valid species by Weighman [28] and Last et al. [29].

*Sequences deposited in BOLD but not publicly available.

**Sequences not deposited in BOLD or GenBank.

Table S2. Primer sequences in 5′-3′ direction, final concentration (C_f, in mM) and annealing temperature (Ta, in °C) utilised in this study for PCR amplification of the two partial mitochondrial fragments Cytochrome Oxidase subunit I (COI) and Nycotinamide dehydrogenase subunit 2 (NADH2). Grey boxes mark the M13-sequencing primer.

Region	Primers 5′-3′	C_f mM	Ta °C	Reference
COI	FishF2: TCGACTAATCATAAAGATATCGGCAC	0.5	54	[24]
	FishR2: ACTTCAGGGTGACCGAAGAATCAGAA			
	VF2_t1: TGTAAAACGACGGCCAGTCAACCAACCACAAAGACATTGGCAC	0.2	52	[25]
	Fish F2_t1: TGTAAAACGACGGCCAGTCGACTAATCATAAAGATATCGGCAC			
	FR1d_t1: CAGGAAACAGCTATGACACCTCAGGGTGTCCGAARAAYCARAA			
	Fish R2_t1: CAGGAAACAGCTATGACACTTCAGGGTGACCGAAGAATCAGAA			
	FF2d: TTCTCCACCAACCACAARGAYATYGG	0.2	52	[25]
	FR1d: CACCTCAGGGTGTCCGAARAAYCARAA			
NADH2	NADH2 Skates FW2: CTACTAATGAACCCCCTCGT	0.2	55	[21]
	NADH2 Skates RV: GCTTTGAAGGCTTTTGGT			
	ND2Met47: TTTTGGGCCCATACC	0.5	54	[26]
	ND2Trp18: GCTTTGAAGGCTTTTGGT			

Table S3. Cases of specimen misidentification. Percentage of similarity (through BOLD Identification Engine) and sampling area of the individuals are presented; the number of misidentified individuals in relation to the total number assessed is provided in parenthesis in the Morphological ID and Barcode ID column.

Morphological ID	Barcode ID	BOLD ID (% Similarity)	Major ocean area, Location	E-Voucher morphological evaluation
Amblyraja radiata (1/20)	*Rajella fyllae* (1/14)	99.69-100	NEA Iceland	*R. fyllae*
Atlantoraja castelnaui (1/19)	*Atlantoraja cyclophora* (1/25)	99.85-100	SWA Brazil	*A. cyclophora*
Atlantoraja cyclophora (1/26)	*Atlantoraja platana* (1/26)	100	SWA Brazil	*A. platana*
Cruriraja sp. (3/4)	*Rajella barnardi* (3/13)	99.5	SEA Angola	No available picture
Cruriraja sp. (1/4)	*Rajella leoparda* (1/22)	99.66	SEA Angola	Juvenile
Cruriraja hulleyi (1/22)	*Raja straeleni* (1/20)	100	SEA South Africa	Juvenile
Dipturus pullopunctatus (2/10)	*Rostroraja alba* (2/14)	100	SEA South Africa	No available picture
Leucoraja fullonica (1/3)	*Dipturus batis* (1/10)	100	NEA Iceland	No available picture
Leucoraja wallacei (1/17)	*Raja straeleni* (1/20)	100	SEA South Africa	Juvenile
Psammobatis extenta (1/1)	*Psammobatis rutrum* (1/10)	100	SWA Brazil	*P. rutrum*
Raja sp. (7/7)	*Raja herwigi – Raja clavata* (7/7)	98.77-98.44	SEA Angola	*Raja sp.*
Rajella caudaspinosa (1/8)	*Cruriraja hulleyi* (1/21)	100	SEA South Africa	No available picture
Rajella caudaspinosa (1/8)	*Rajella barnardi* (1/13)	99.5	SEA South Africa	*R. barnardi*
Rajella caudaspinosa (2/8)	*Rajella leoparda* (2/22)	99.66	SEA South Africa	*R. leoparda*
Rajella dissimilis (1/7)	*Raja straeleni* (1/20)	100	SEA South Africa	Juvenile
Rajella leoparda (1/28)	*Cruriraja hulleyi* (1/21)	100	SEA South Africa	No available picture
Rajella leoparda (1/28)	*Raja straeleni* (1/20)	100	SEA South Africa	Juvenile
Rajella leoparda (4/28)	*Rajella dissimilis* (4/6)	100	SEA South Africa	*R.dissimilis*
Rioraja agassizii (2/12)	*Atlantoraja platana* (2/26)	100	SWA Brazil	*A. platana*
Rostroraja alba (1/16)	*Cruriraja hulleyi* (1/21)	100	SEA South Africa	No available picture
Rostroraja alba (2/16)	*Raja straeleni* (2/20)	99.33-99.5	SEA South Africa	No available picture

Table S4. Sequence diversity for Cytochrome Oxidase subunit I for Atlantic skates included in the present study. For each taxon, the following fields are presented: number of individuals (N), maximum intraspecific genetic distance (Max Intra-Sp), distance to nearest neighbour (Distance to NN), number of haplotypes (nH), haplotype diversity (Hd), nucleotide diversity (π), sampling locations, Barcode Index Number (BIN) obtained from BOLD (blue text refers to Unique BIN, red text refers to Discordant BIN, black text refers to Concordant BIN), and abbreviation ID used in phylogenetic analyses. IUCN Categories are also indicated: least concern (LC), near threatened (NT), vulnerable (VU), endangered (EN), critically endangered (CR), not evaluated (NE).

Species	N	Max Intra-Sp	Distance to NN	Geographical areas	IUCN category	BIN	Elasmo-Atl/ Total number in BOLD	ID
ARHYNCHOBATIDAE								
Atlantoraja castelnaui	18	0.17	8.33	SWA Brazil	CR	BOLD:AAB4961	18/30	Atcas1
Atlantoraja cyclophora	25	1.03	4.49	SWA Brazil	EN	BOLD:AAB1882	25/35	Atcyc1
Atlantoraja platana	26	0.34	4.49	SWA Brazil	EN	BOLD:AAB1884	26/29	Atpla1
Bathyraja richardsoni	6	0.37	3.04	NEA Mid-Atlantic Ridge	LC	BOLD:AAA8067	6/10	Bric1
Bathyraja spinicauda	10	0.52	3.04	NEA Iceland	NT		10/225	Bspi1
Psammobatis rutrum	10	0.52	10.94	SWA Brazil	LC	BOLD:AAE0763	10/10	Prut1
Rioraja agassizii	10	0.69	9.3	SWA Brazil	VU	BOLD:AAB1883	10/29	Raga1
GURGESIELLIDAE								
Cruriraja hulleyi	21	0.52	15.78	SEA South Africa	LC	BOLD:AAB7055	21/30	Chul1
RAJIDAE								
Amblyraja hyperborea	13	0.34	2.69	NEA Iceland	LC	BOLD:ABZ5141	13/207	Ahyp1
Amblyraja radiata	19	1.20	2.69	NEA Iceland	VU	BOLD:AAA4500	19/259	Arad1
Dipturus pullopunctatus	9	0.00	6.14	SEA South Africa	LC	BOLD:AAD3653	9/38	Dpul1
Dipturus batis	10	0.69	3.61	NEA Iceland; Western English Channel	CR	BOLD:AAC0752	10/33	Dbat1
Dipturus intermedius	5	0.00	3.61	NEA Western English Channel	NE	BOLD:AAA8675	5/41	Dint1
Dipturus nidarosiensis	1	N/A	5.81	CEA Morocco	NT	BOLD:AAE1030	1/14	Dnid1
Leucoraja fullonica	2	0.00	6.06	NEA Celtic Sea	VU	BOLD:AAE3437	2/8	Lful1
Leucoraja naevus	2	0.52	2.39	NEA Celtic Sea	LC	BOLD:AAB0817	2/70	Lnae1
Leucoraja wallacei	16	0.69	2.39	SEA South Africa	VU	BOLD:ABZ1536	16/23	Lwal1
Raja brachyura	2	0.52	4.59	NEA Celtic Sea; Portugal	NT	BOLD:AAA4358	2/59	Rbra1
Raja clavata	14	0.70	0.91	NEA Celtic Sea; Irish Sea; North Sea; Norwegian Sea	NT	BOLD:ACF2419	16/143	Rcla1
Raja maderensis	2	0.00	0.91	CEA Madeira	VU			Rmad1

Raja microocellata	4	0.17	4.59	NEA Bristol Channel, Bay of Biscay	NT	BOLD:AAE7627	4/7	Rmic1
Raja cf. miraletus Angola 1	13	0.64	2.55	SEA Angola	LC	BOLD:ABZ6837	13/13	Rmir10
Raja cf. miraletus Angola 2	4	0.00	3	SEA Angola		BOLD:AAA4475	4/4	Rmir5
Raja cf. miraletus Senegal	4	0.41	5.15	CEA Cape Verde		BOLD:AAZ1588	4/5	Rmir1
Raja ocellifera	13	0.64	2.55	SEA South Africa	EN	BOLD:AAA4359	13/20	Rmir32
Raja montagui	63	0.34	5.26	NEA Celtic Sea; Irish Sea	LC	BOLD:AAA4356	63/110	Rmon1
Raja sp.	7	0.00	1.76	SEA Angola		BOLD:AAA4361	7/7	Rsp1
Raja straeleni	20	1.03	1.46	SEA South Africa	DD	BOLD:ACF2420	20/26	Rstr1
Raja undulata	2	0.17	6.78	NEA Bay of Biscay; Western English Channel	EN	BOLD:AAL0858	2/12	Rund1
Rajella barnardi	13	0.34	0.82	SEA Angola; South Africa	LC	BOLD:AAA4360	35/37	Rlbar1
Rajella leoparda	22	0.34	0.82	SEA Angola; South Africa	LC			Rlleo1
Rajella bathyphila	3	0.00	2.78	NEA Iceland	LC	BOLD:ABZ5259	3/20	Rlbat1
Rajella caudaspinosa	4	0.34	2.49	SEA South Africa	LC	BOLD:ADK9017	4/4	Rlcau1
Rajella dissimilis	6	0.34	1.43	SEA South Africa	LC	BOLD:ACF1708	6/9	Rldis1
Rajella fyllae	14	0.86	2.49	NEA Iceland	LC	BOLD:AAA4502	14/79	Rlfyl1
Rajella kukujevi	4	0	1.43	NEA mid-Atlantic Ridge; Bay of Biscay; Ireland; Scotland	LC	BOLD:ACF1707	4/10	Rlkuk1
Rajella lintea	1	0	3.96	NEA Iceland	LC	BOLD:AAD0011	1/17	Rllin
Rostroraja alba	14	0	11.8	SEA South Africa	EN	BOLD:AAB8691	14/26	Ralb1

Table S5. Substitution saturation test measuring whether the observed index of substitution saturation (Iss) is significantly lower than critical Iss value (Iss.c), with different numbers of operational taxonomic units (Num.OTU) for COI and NADH2.

Marker	Num.OTU	Iss	Iss.c	T	DF	P
NADH2	4	0.315	0.806	21.246	443	0.000
	8	0.316	0.767	18.828	443	0.000
	16	0.317	0.746	18.013	443	0.000
	32	0.324	0.720	16.730	443	0.000
COI	4	0.387	0.802	13.818	255	0.000
	8	0.376	0.761	12.926	255	0.000
	16	0.378	0.737	12.622	255	0.000
	32	0.384	0.712	11.907	255	0.000

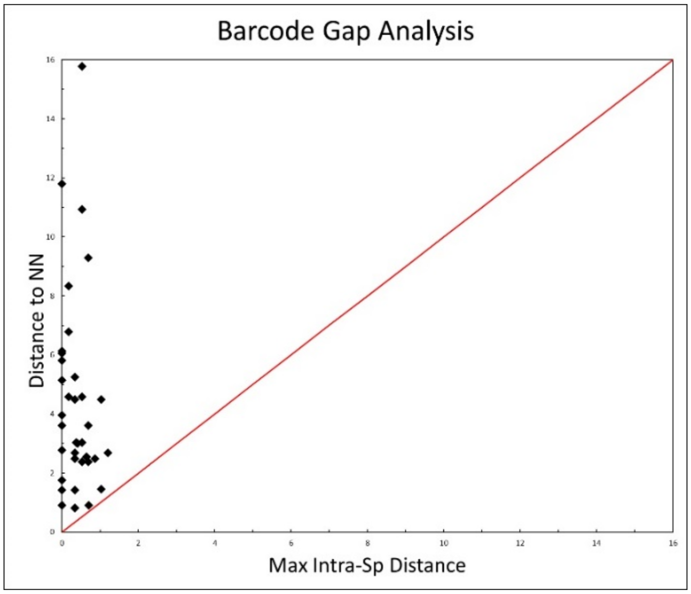

Figure S1. Maximum intraspecific distance plotted against the minimum inter-specific (Nearest Neighbour) distances (p-distance values) for the COI barcode sequences of the 38 MOTUs. The 1:1 equivalence (straight line) is indicated.

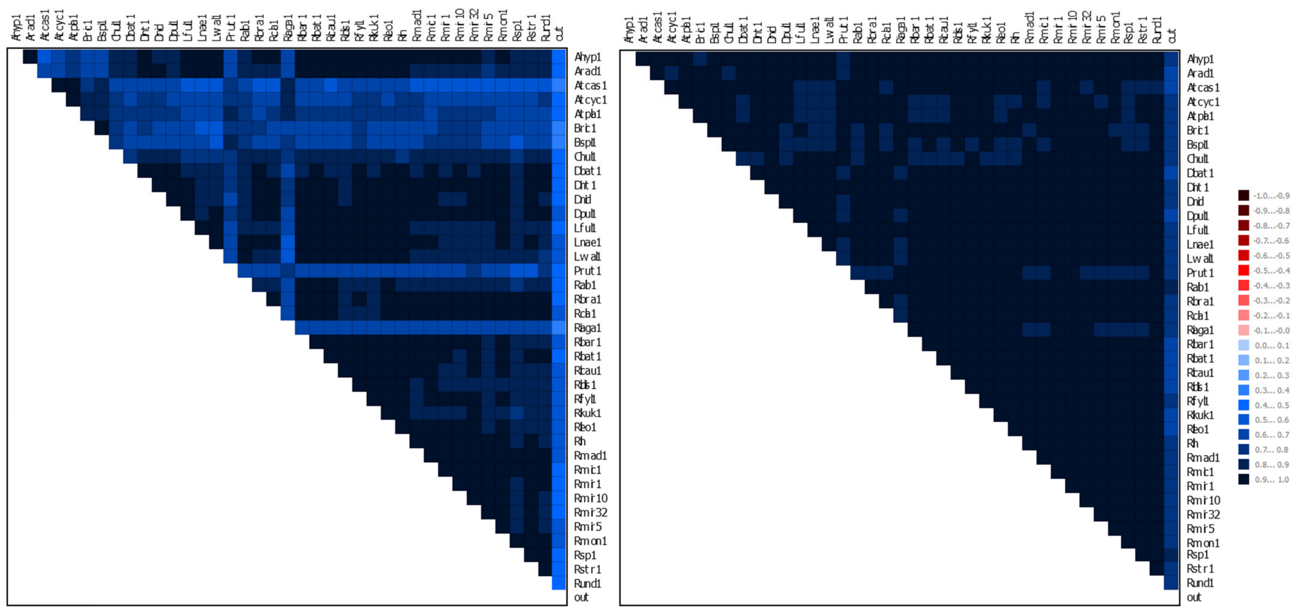

(a) (b)

Figure S2. AliGROOVE analysis based on 38 mitochondrial sequences of Atlantic skates. (a) NADH2; (b) COI. The mean similarity score between sequences is represented by a coloured square. Taxon names are consistent with the column "ID" in Table S4. The outgroup (out) is represented by homologous sequence of *Squalus acanthias* (GenBank code: NC_002012).

(a)

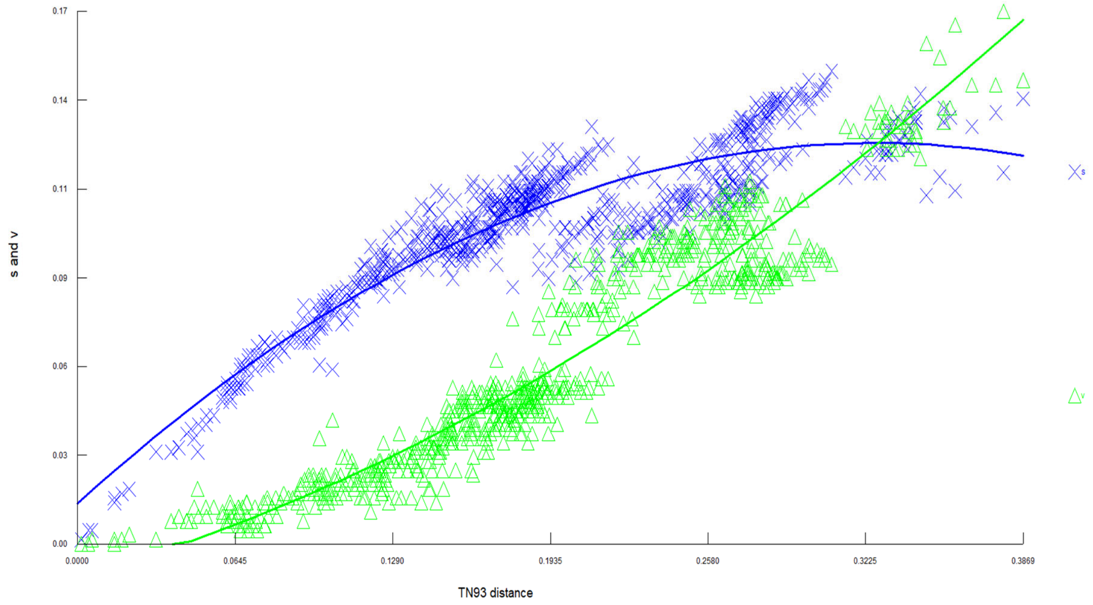

(b)

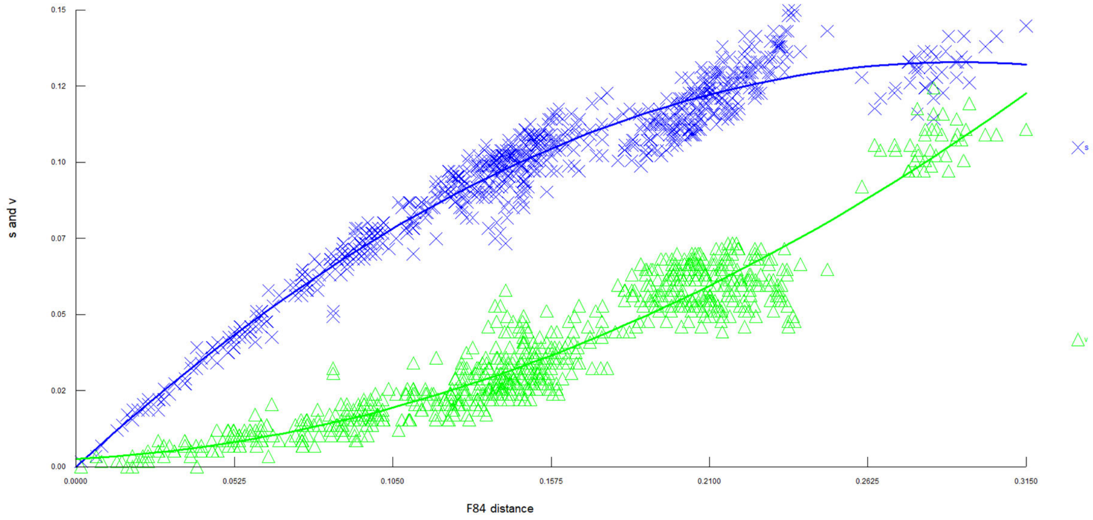

Figure S3. Plot of the number of transitions (s, blue cross) and transversions (v, green triangle) vs. genetic distance. The curves show the trends of variance of transitions and transversions with increasing genetic distance. a) NADH2; b) COI.

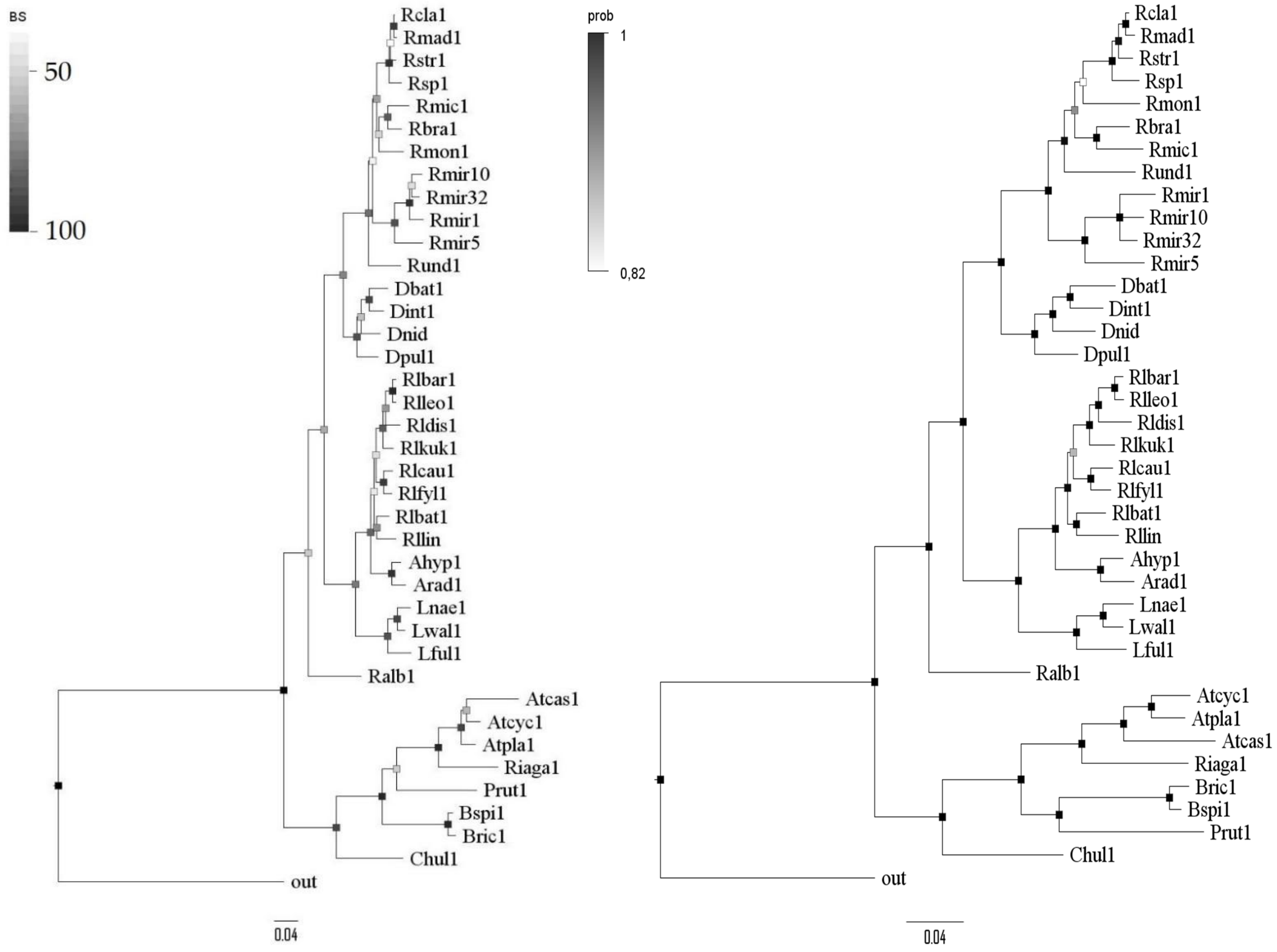

(a) (b)

Figure S4. Maximum-likelihood (a) and Bayesian (b) phylogenetic trees based on concatenated NADH2 and COI datasets. Statistical measures of Maximum-likelihood bootstrap (BS) and Bayesian posterior probability (prob) support are shown at each node. A distance scale bar is given. Names at tips are consistent with the column "ID" in Table S4. The outgroup (out) is represented by homologous sequence of *Squalus acanthias* (GenBank code: NC_002012).

References

1. Mabragaña, E.; Astarloa, J.M.D. de; Hanner, R.; Zhang, J.; Castro, M.G. DNA Barcoding Identifies Argentine Fishes from Marine and Brackish Waters. *PLOS ONE* **2011**, *6*, e28655, doi:10.1371/journal.pone.0028655.
2. Coulson, M.W.; Denti, D.; Guelpen, L.V.; Miri, C.; Kenchington, E.; Bentzen, P. DNA Barcoding of Canada's Skates. *Mol. Ecol. Resour.* **2011**, *11*, 968–978, doi:https://doi.org/10.1111/j.1755-0998.2011.03034.x.
3. Lynghammar, A.; Christiansen, J.S.; Griffiths, A.M.; Fevolden, S.-E.; Hop, H.; Bakken, T. DNA Barcoding of the Northern Northeast Atlantic Skates (Chondrichthyes, Rajiformes), with Remarks on the Widely Distributed Starry Ray. *Zool. Scr.* **2014**, *43*, 485–495, doi:https://doi.org/10.1111/zsc.12064.
4. Lago, F.C.; Vieites, J.M.; Espiñeira, M. Development of a FINS- Based Method for the Identification of Skates Species of Commercial Interest. *Food Control* **2012**, *24*, 38–43, doi:10.1016/j.foodcont.2011.08.034.
5. Knebelsberger, T.; Landi, M.; Neumann, H.; Kloppmann, M.; Sell, A.F.; Campbell, P.D.; Laakmann, S.; Raupach, M.J.; Carvalho, G.R.; Costa, F.O. A Reliable DNA Barcode Reference Library for the Identification of the North European Shelf Fish Fauna. *Mol. Ecol. Resour.* **2014**, *14*, 1060–1071, doi:https://doi.org/10.1111/1755-0998.12238.
6. Ribeiro, A. de O.; Caires, R.A.; Mariguela, T.C.; Pereira, L.H.G.; Hanner, R.; Oliveira, C. DNA Barcodes Identify Marine Fishes of São Paulo State, Brazil. *Mol. Ecol. Resour.* **2012**, *12*, 1012–1020, doi:https://doi.org/10.1111/1755-0998.12007.
7. Ferrette, B.L. da S.; Domingues, R.R.; Rotundo, M.M.; Miranda, M.P.; Bunholi, I.V.; De Biasi, J.B.; Oliveira, C.; Foresti, F.; Mendonça, F.F. DNA Barcode Reveals the Bycatch of Endangered Batoids Species in the Southwest Atlantic: Implications for Sustainable Fisheries Management and Conservation Efforts. *Genes* **2019**, *10*, 304, doi:10.3390/genes10040304.
8. Smith, P.J.; Steinke, D.; Mcveagh, S.M.; Stewart, A.L.; Struthers, C.D.; Roberts, C.D. Molecular Analysis of Southern Ocean Skates (*Bathyraja*) Reveals a New Species of Antarctic Skate. *J. Fish Biol.* **2008**, *73*, 1170–1182, doi:10.1111/j.1095-8649.2008.01957.x.
9. Spies, I.B.; Gaichas, S.; Stevenson, D.E.; Orr, J.W.; Canino, M.F. DNA-Based Identification of Alaska Skates (Amblyraja, Bathyraja and Raja: Rajidae) Using Cytochrome c Oxidase Subunit I (CoI) Variation. *J. Fish Biol.* **2006**, *69*, 283–292, doi:https://doi.org/10.1111/j.1095-8649.2006.01286.x.
10. Cannas, R.; Follesa, M.C.; Cabiddu, S.; Porcu, C.; Salvadori, S.; Iglésias, S.P.; Deiana, A.M.; Cau, A. Molecular and Morphological Evidence of the Occurrence of the Norwegian Skate Dipturus Nidarosiensis (Storm, 1881) in the Mediterranean Sea. *Mar. Biol. Res.* **2010**, *6*, 341–350, doi:10.1080/17451000903428496.
11. Costa, F.O.; Landi, M.; Martins, R.; Costa, M.H.; Costa, M.E.; Carneiro, M.; Alves, M.J.; Steinke, D.; Carvalho, G.R. A Ranking System for Reference Libraries of DNA Barcodes: Application to Marine Fish Species from Portugal. *PLOS ONE* **2012**, *7*, e35858, doi:10.1371/journal.pone.0035858.
12. Rodríguez-Cabello, C.; Pérez, M.; Sánchez, F. New Records of Chondrichthyans Species Caught in the Cantabrian Sea (Southern Bay of Biscay). *J. Mar. Biol. Assoc. U. K.* **2013**, *93*, 1929–1939, doi:10.1017/S0025315413000271.
13. Cariani, A.; Messinetti, S.; Ferrari, A.; Arculeo, M.; Bonello, J.J.; Bonnici, L.; Cannas, R.; Carbonara, P.; Cau, A.; Charilaou, C.; et al. Improving the Conservation of Mediterranean Chondrichthyans: The ELASMOMED DNA Barcode Reference Library. *PLOS ONE* **2017**, *12*, e0170244, doi:10.1371/journal.pone.0170244.
14. Ramírez-Amaro, S.; Ordines, F.; Puerto, M.Á.; García, C.; Ramon, C.; Terrasa, B.; Massutí, E. New Morphological and Molecular Evidence Confirm the Presence of the Norwegian Skate Dipturus Nidarosiensis (Storm, 1881) in the Mediterranean Sea and Extend Its Distribution to the Western Basin. *Mediterr. Mar. Sci.* **2017**, *18*, 251–259, doi:10.12681/mms.1950.
15. Carbonara, P.; Cannas, R.; Donnaloia, M.; Melis, R.; Porcu, C.; Spedicato, M.T.; Zupa, W.; Follesa, M.C. On the Presence of Dipturus Nidarosiensis (Storm, 1881) in the Central Mediterranean Area. *PeerJ* **2019**, *7*, doi:10.7717/peerj.7009.
16. Serra-Pereira, B.; Moura, T.; Griffiths, A.M.; Gordo, L.S.; Figueiredo, I. Molecular Barcoding of Skates (Chondrichthyes: Rajidae) from the Southern Northeast Atlantic. *Zool. Scr.* **2011**, *40*, 76–84, doi:https://doi.org/10.1111/j.1463-6409.2010.00461.x.
17. Landi, M.; Dimech, M.; Arculeo, M.; Biondo, G.; Martins, R.; Carneiro, M.; Carvalho, G.R.; Brutto, S.L.; Costa, F.O. DNA Barcoding for Species Assignment: The Case of Mediterranean Marine Fishes. *PLOS ONE* **2014**, *9*, e106135, doi:10.1371/journal.pone.0106135.
18. Vella, A.; Vella, N.; Schembri, S. A Molecular Approach towards Taxonomic Identification of Elasmobranch Species from Maltese Fisheries Landings. *Mar. Genomics* **2017**, *36*, 17–23, doi:10.1016/j.margen.2017.08.008.
19. Ramírez-Amaro, S.; Ordines, F.; Picornell, A.; Castro, J.A.; Ramon, C.; Massutí, E.; Terrasa, B. The Evolutionary History of Mediterranean Batoidea (Chondrichthyes: Neoselachii). *Zool. Scr.* **2018**, *47*, 686–698, doi:https://doi.org/10.1111/zsc.12315.
20. Steinke, D.; Connell, A.D.; Hebert, P.D.N. Linking Adults and Immatures of South African Marine Fishes. *Genome* **2016**, doi:10.1139/gen-2015-0212.
21. Ferrari, A.; Tinti, F.; Maresca, V.B.; Velonà, A.; Cannas, R.; Thasitis, I.; Costa, F.O.; Follesa, M.C.; Golani, D.; Hemida, F.; et al. Natural History and Molecular Evolution of Demersal Mediterranean Sharks and Skates Inferred by Comparative Phylogeographic and Demographic Analyses. *PeerJ* **2018**, *6*, e5560, doi:10.7717/peerj.5560.
22. Ball, R.E.; Serra-Pereira, B.; Ellis, J.; Genner, M.J.; Iglésias, S.; Johnson, A.F.; Jones, C.S.; Leslie, R.; Lewis, J.; Mariani, S.; et al. Resolving Taxonomic Uncertainty in Vulnerable Elasmobranchs: Are the Madeira Skate (Raja Maderensis) and the Thornback Ray (Raja Clavata) Distinct Species? *Conserv. Genet.* **2016**, *17*, 565–576, doi:10.1007/s10592-015-0806-1.
23. Last; Séret A New Eastern Central Atlantic Skate Raja Parva Sp. Nov. (Rajoidei: Rajidae) Belonging to the Raja Miraletus Species Complex. *Zootaxa* **2016**, *4147*, 477–489, doi:10.11646/zootaxa.4147.4.8.
24. Ward, R.D.; Zemlak, T.S.; Innes, B.H.; Last, P.R.; Hebert, P.D.N. DNA Barcoding Australia's Fish Species. *Philos. Trans. R. Soc. B Biol. Sci.* **2005**, *360*, 1847–1857, doi:10.1098/rstb.2005.1716.

25. Ivanova, N.V.; Zemlak, T.S.; Hanner, R.H.; Hebert, P.D.N. Universal Primer Cocktails for Fish DNA Barcoding. *Mol. Ecol. Notes* **2007**, *7*, 544–548, doi:https://doi.org/10.1111/j.1471-8286.2007.01748.x.
26. Sandoval-Castillo, J.; Rocha-Olivares, A. Deep Mitochondrial Divergence in Baja California Populations of an Aquilopelagic Elasmobranch: The Golden Cownose Ray. *J. Hered.* **2011**, *102*, 269–274, doi:10.1093/jhered/esr004.
27. Gabbanelli, V.; Díaz de Astarloa, J.M.; Gonzalez-Castro, M.; Vazquez, D.M.; Mabragaña, E. Almost a Century of Oblivion: Integrative Taxonomy Allows the Resurrection of the Longnose Skate Zearaja Brevicaudata (Marini, 1933) (Rajiformes; Rajidae). C. R. Biol. **2018**, 341, 454–470, doi:10.1016/j.crvi.2018.10.002.
28. Weigmann, S. Annotated Checklist of the Living Sharks, Batoids and Chimaeras (Chondrichthyes) of the World, with a Focus on Biogeographical Diversity. *J. Fish Biol.* **2016**, *88*, 837–1037, doi:https://doi.org/10.1111/jfb.12874.
29. Last, P.; Weigmann, S.; Yang, L. Changes to the nomenclature of the skates (Chondrichthyes: Rajiformes). In: Last, P.R. & Yearsley, G.K. (Eds.) *Rays of the World: Supplementary information*, CSIRO Australian National Fish Collection: Canberra, Australia, 2016, CSIRO Special Publication: 11–34
30. White, W.T.; Fricke, R. *Raja Mauritaniensis*: A replacement name for *Raja Africana* Capapé, 1977 (Rajiformes: Rajidae), a junior homonym of *Raja Africana* Bloch & Schneider, 1801 (Myliobatiformes: Dasyatidae. *Zootaxa* **2021**, *4970*, 399–400, doi:https://doi.org/10.11646/zootaxa.4970.2.12.
